# Cognitive liberty and the psychedelic humanities

**DOI:** 10.3389/fpsyg.2023.1128996

**Published:** 2023-05-04

**Authors:** Osiris González Romero

**Affiliations:** Department of History, University of Saskatchewan, Saskatoon, SK, Canada

**Keywords:** psychedelics, humanities, cognitive liberty, philosophy, history, anthropology

## Abstract

This research aims to conceptualize cognitive liberty and the psychedelic humanities by examining their constitutive elements. The importance of this study lies in the fact that it is widespread to talk about psychedelic science nowadays, but there is a significant gap in the research. For instance, the role and importance of the humanities need to be acknowledged. Regarding cognitive liberty, this research considers that people have the right to use or refrain from using emerging neurotechnologies and psychedelics. People’s freedom of choice *vis-à-vis* these technologies must be protected, in particular with regard to coercive and non-consensual uses. Firstly, an analysis will be carried out of the constitutive elements of cognitive liberty, especially within the context of a philosophical approach. Secondly, this research will address some arguments for the philosophical uses of psychedelics. Finally, this paper will discuss the scope and significance of psychedelic humanities as a vein of research. Cognitive liberty is a crucial concept for the psychedelic humanities, likely to broaden our understanding of consciousness studies and reflect on ethical and social issues related to scientific research. Cognitive liberty is an update of freedom of thought according to the challenges of the 21st century. In addition, this paper will highlight the possible philosophical uses of psychedelic substances to broaden the research scope since, at present, the ritual and therapeutic uses of psychedelics have the most significant legitimacy. Recognition of philosophical uses demonstrates that learning from non-clinical uses of psychedelics is possible. The psychedelic humanities represent an underexplored avenue of research that can contribute to a better understanding of the interplay between science and culture.

## 1. Introduction

The war on drugs and the associated prohibition policies have undermined freedom of thought for several decades, not only individual freedom but also the cognitive liberty of research communities and institutions worldwide. The war on drugs “is not a war on pills, powder, plants, and potions, it is a war on mental states — a war on consciousness itself — how much, what sort we are permitted to experience, and who gets to control it” (Boire, 1999, 6). Furthermore, the war on drugs has encouraged prohibitionist policies that have disproportionately affected some regions and communities more than others. Recent decriminalization efforts with cannabis and psychedelics have begun challenging this logic, revealing other consequences of historical prohibitions, i.e., deliberate misinformation, propaganda, racialization and massive incarceration. The war on drugs has interrupted scientific progress, while criminal activities related to weapons trafficking, substance adulteration, struggles for territory, and strengthening of the black market have given rise to organized crime networks and undermined cognitive liberty and scientific research. In fact, it can be argued that the war on drugs suits two sectors more than anything else: “the drug traffickers because the illicit trade is at the heart of their business and their vast profits, and the governments, especially the U.S., because with this war, they justify massive budgets, expansion of security forces, and ‘legitimize’ the control and repression of their people inside and outside their territory” (Brooks, 2023).

This research highlights the crucial role of the humanities and social sciences (philosophy, history, anthropology, literature, and women’s studies) in promoting a transdisciplinary approach and a more contextualized understanding of psychedelic studies. For instance, analyzing therapeutic and philosophical uses, decolonizing efforts, drug tourism, and harm reduction against the backdrop of studies in cognitive liberty. Fortunately, since the beginning of the 21st century, this prohibition has started to change gradually. The research about psychedelics such as Dimethyltryptamine (DMT), Lysergic Acid Diethylamide (LSD), Methylenedioxide Methamphetamine (MDMA), psilocybin, and mescaline has increased under the umbrella of the psychedelic renaissance (Sessa, 2012, 159; Letheby, 2021, 8–13; Gomez-Escolar, 2022). At the same time, some legislation, primarily related to cannabis and psychedelics, has changed in countries such as the Netherlands, Australia, Canada, USA, Portugal, Uruguay, Perú, Brazil, Colombia and Mexico, opening new avenues of research, as well as stimulating older cultural networks of knowledge concerning drug use and abuse; for example, psychedelics are not free from risks. Due to these risks, examining the evidence provided by scientific research is necessary to achieve a more balanced and informed perspective, avoiding biases as much as possible.

To begin this analysis, it is necessary to highlight the primary uses of psychedelics, which the scientific literature recognizes mainly: (1) therapeutic uses, (2) religious and spiritual uses, (3) creative uses, and (4) hedonistic or adult uses. This research clarifies the scope of (5) philosophical uses within the framework of psychedelic humanities. A philosophical approach considers the psychedelic experience as a source of knowledge, not only a series of hallucinations. What kind of knowledge? This paper is an attempt to explain this intricate matter clearly.

The rise of neuroscience and the increase of technologies capable of affecting or monitoring cognition, such as functional magnetic resonance imaging (fMRI), transcranial magnetic stimulation (TMS), brain implants, brain-machine interfaces, or the implementation of biochemistry and neuropsychopharmacology to enhance or modify human cognition caused new philosophical dilemmas. This development of neuroscience requires an update of our conception of freedom of thought to face that transformation successfully. In this context, some scholars considered recognizing the right of cognitive liberty as the first step to avoiding mental manipulation (Sententia, 2004, 221–228; Ienca, 2017, 10–11; Weissenbacher, 2018, 1–11). Cognitive liberty is every person’s fundamental right to think independently, use the full spectrum of his or her mind, and have autonomy over their brain chemistry. This fundamental right implies some ethical issues which are necessary to consider. For example: “Cognitive liberty concerns the ethics and legality of safeguarding one’s own thought processes, and by necessity, one’s electrochemical brain states. The individual, not corporate and government interests, should have sole jurisdiction over the control and/or modulation of his and her brain states and mental processes” (Sententia, 2004, 223).

Implementing some neurotechnologies mentioned above gave rise to new branches of knowledge, such as neurotheology, neuromarketing, neuropolitics, and neuromodulation. They are directly related to freedom of choice and the transformation of beliefs. The emergence of this power challenged our previous frameworks on mental autonomy, which require an update to guarantee the right of cognitive liberty under the premises of free, prior, and informed consent. An analytical approach regarding this fundamental right shows two related principles: “The first is that persons have the right to use or refrain from using emerging neurotechnologies. The second one is that people deserve protection from such technologies coercive and unconsented use. A person has the right of self-determination, defined as the right to change his or her mind and choose how change occurs” (Weissenbacher, 2018, 8). It is possible to appreciate that freedom of choice and self-determination are two constitutive elements of cognitive liberty.

Cognitive liberty involves ethical issues, such as freedom of thought, absence of interference, mental autonomy, responsibility, and human rights. For example, cultural, indigenous, women’s, and health rights (Fletcher, 2012, 225–242). Nevertheless, what exactly is meant by the term’ cognitive liberty in the context of the psychedelic humanities? Law lecturer and cognitive liberty advocate Charlotte Walsh argues: “Cognitive liberty is in one sense synonymous with freedom of thought, yet more precisely evokes the idea that this should be read to acknowledge the fact that individuals should have the right to autonomous self-determination over their own brain chemistry, a right that is currently infringed by the prohibition of psychedelics” (Walsh, 2016, 83). This definition sheds light on the other two constitutive elements of cognitive liberty: freedom of thought and autonomy.

The role of the humanities is critically important to understand better some social implications embedded in the therapeutic uses of psychedelics, but also to reflect from a cross-cultural perspective on oriented psychedelic experiences, i.e., psychedelics can participate in a significant revaluation of liberty. But also, to critically analyze the medicalization attached to psychedelic capitalism (Noorani, 2019, 34–39). New scholarship in psychedelic studies is beginning to recognize the philosophical and therapeutic uses of psychedelics and their consequences in a 21st-century context (Letheby, 2021, 62–80) and to more fully recognize the power relations and social issues embedded in the use of psychedelics (Hauskeller et al., 2022, 107–132). Based on this scholarship, this paper aims to analyze the scope of cognitive liberty within the context of psychedelic humanities.

This article understands by the psychedelic humanities, for instance, the philosophy of psychedelics, the history of medicine, neuroethics, and medical anthropology (Roberts, 2017, 102–105). This paper builds on this scholarship specifically by interrogating the philosophical framework grounded in the enhancement of cognitive liberty and the potential transformations of the psychedelic experience because “several influential figures saw psychedelics and their power to radically question ontological certainties as having an inherently revolutionary potential” (Elcock, 2013, 296). Moreover, some authors consider it possible to discuss the emancipatory uses of psychedelics (Hendlin, 2022). For instance: “Peyotism is an attempt by American Indians not only to cope with contemporary social and economic conditions, but also to master and ultimately to transform them” (Wagner, 1975, 204). An interdisciplinary approach is required to face the possible objections and misunderstandings related to the different uses of psychedelics and clarify their transformative character in different cultures and social classes. Oriented psychedelic experiences can contribute to overcoming cultural biases and building different narratives to achieve a shift within the hegemonic public policies.

## 2. Constitutive elements of cognitive liberty

The relevance of this research lies in analyzing a concept (liberty) that is considered the foundation of Ethics and Politics. This section aims to display a brief overview of the constitutive elements of cognitive liberty. An analytical approach is crucial to a better understanding of their contemporary significance for the psychedelic humanities, mainly because philosophy and psychedelics help expand consciousness (Sjöstedt-Hughes, 2016). As is possible to see, there is a contemporary approach regarding cognitive liberty and mental autonomy, which has been taken up in psychedelic studies during the 21st century by some scholars such as Boire (1999), Walsh (2010, 2014, 2016), and Davis (2022).

The social relevance of cognitive liberty is not purely historical or speculative. In this regard, it is very significant to recognize that some sensitive political issues, such as the growth of far-right movements and hate speech (Langlitz, 2020a; Pace and Devenot, 2021), as well as the rise of populism and migration, have challenged the social significance of liberty in the contemporary world and psychedelic studies. Furthermore, the self-contradictions and ambiguities embedded in the concept of liberty –mainly implemented as a domination tool- have undermined its legitimacy due to the failures and injustices caused by neo-liberal and technocratic regimes. For example, libertarianism reshaped the concept of liberal autonomy into “consumer sovereignty” (Davis, 2022, 92), assuming that a free-market society must prioritize medicalization, the interests of pharmaceutical companies, the opening of new markets, the rise of psychedelic CEOS and the wishes and expectations of consumers (Fernández, 2022). One of the side effects of this degraded consumerist view of autonomy is the banalization of the psychedelic experience, but also the paradoxical use of liberty as a mechanism of control (González Romero, 2022).

To overcome some paradoxes and social contradictions attached to the psychedelic renaissance, it is necessary to reflect critically on liberty and update its meaning to the present needs. For instance, in the realm of psychedelic humanities, the significance of cognitive liberty is related to consciousness. “The right to control one’s own consciousness is the quintessence of freedom. If freedom is to mean anything, it must mean that each person has an inviolable right to think for him or herself. It must mean, at a minimum, that each person is free to direct one’s own consciousness, underlying mental processes, beliefs, opinions, and worldview” (Boire, 1999, 4). It implies the right to use psychedelics to enhance cognition and strengthen self-reflection and creativity. Cognitive liberty can contribute to consciousness studies by challenging the attempts to control it embedded in prohibitionist policies and within the commodification encouraged by psychedelic capitalism. Furthermore, the scope of cognitive liberty involves challenging topics such as the forced administration of psychotropics by states, endocrine disruptor exposure, euthanasia, and the right to die.

In other words, in the field of psychedelic humanities, the meaning of cognitive liberty is the right of everyone to think independently and autonomously—the use of the full power of the mind and the engagement in multiple ways of thought. Mental autonomy implies the right to self-determine one’s own brain chemistry. The connection between cognitive liberty and psychedelics could be summarized using the well-known frameworks of negative liberty developed by Mill (1982) and positive liberty coined by Berlin (2002/1969, 166). Regarding cognitive liberty, it is possible to talk about: (1) the right to refuse, i.e., absence of interference or negative liberty, and (2) the right to use, i.e., the enhancement of cognition or positive liberty. This neuroenhancement is one of the critical traits advocated by psychedelic activism. A popular version of this topic was stated by Timothy Leary some decades before the term cognitive liberty was coined. The Two Commandments for the Molecular Age states: “I.-Thou shalt not alter the consciousness of thy fellow man, and II.-Thou shalt not prevent thy fellow man from altering his own consciousness” (Leary, 1998, 95).

Summarizing, cognitive liberty involves at least four philosophical meanings: (1) freedom of choice, (2) freedom of religion, (3) self-determination, and (4) freedom of thought. Each one has specific implications in the field of psychedelic humanities, which is necessary to explain to avoid misunderstandings and hasty generalizations. So, prohibition policies undermine all of them, not only in a theoretical way but also represent a lack of recognition of fundamental human rights. The first three philosophical meanings will be examined quickly in what follows, and the last one in the section devoted to the philosophy of psychedelics.

### 2.1. Freedom of choice (*Liberum arbitrium*)

Freedom of choice goes beyond the therapeutic uses of psychedelics. Also, it involves the development of human faculties, the enhancement of cognitive skills, the achievement of psychological insights, and gender issues, i.e., choosing one’s gender or identity. The choices mainly constitute the personality, and interfering with these choices thus threatens the free development of personality. In the philosophical realm, freedom of choice expresses itself through the figure of dilemma, i.e., the possibility of choosing between A or B. Every dilemma has a lot of emotional, rational, and social implications, and the role of psychedelic humanities, especially philosophy, is to support individual and collective choices with truthful information and accurate frameworks. Our daily life is full of dilemmas, so it is crucial to consider what is beneficial or not for our body and mind. Concerning the psychedelic realm, the following statement is helpful to clarify the scope of some dilemmas involved with freedom of choice:

From the skin inward is my jurisdiction, is it not? I choose what may or may not cross that border. Here I am the Customs Agent. I am the Coast Guard. I am the sole legal and spiritual Government of this territory, and only the laws I choose to enact within myself are applicable […] What I think? Where I focus my awareness? *What biochemical reactions I choose to cause within the territorial boundaries of my own skin are not subject to the beliefs, morals, laws, or preferences of any other person! I am a sovereign state*, and I feel that my borders are more sacred than the politically drawn boundaries of any country (Shulgin and Shulgin, 1991, pp. 449–450).

This quote clearly shows the scope of freedom of choice and human rights within the psychedelic experience and sheds light on the links between self-consciousness and self-determination. Firstly, this statement represents a clear example of individual freedom regarding prohibitionist policy. It represents freedom of choice concerning which psychoactive substances will cross the border of body and consciousness, but also freedom of choice regarding which norms or laws (*nomoi*) are applicable (Davis, 2022). Secondly, this statement addresses the concept of individual sovereignty, tracing an analogy between the borders of individual consciousness and a country’s borders. Freedom of choice and self-determination usually come together, not only due to the psychedelic experience but also related to bodily autonomy. For example, following the renewed interest in sexual violence, the curtailment of abortion rights in the United States has come with a revived interest in the political necessity of bodily autonomy familiar from the second wave of feminism (Hewitt, 2019; Davis, 2022, 91).

Freedom of choice and bodily autonomy are constitutive elements of cognitive liberty. Both have a crucial significance in developing psychedelic humanities in different branches, such as choosing the identity, appropriate psychedelic assisted therapy and implementing public policies based on harm reduction and the management of pleasure, which is an overlooked issue. As it is possible to appreciate, prohibition policies undermine a wide array of civil liberties, for example, the free development of personality, the right to health, and indigenous rights. Concerning the interplay between prohibition and human rights policies, Walsh (2010, 2014, 2016) examined some conflicts and paradoxes within the freedom of choice. Through a detailed analysis of some concrete examples and tensions between the European Convention on Human Rights (ECHR), especially article 9, and the Misuse of Drugs Act (1971), which drives drug policy in the UK. With this framework in mind, she addressed three complex issues: (1) self-medication, (2) freedom of religion, and (3) the right to explore our self-consciousness.

Self-medication requires an examination because it focuses mainly on the case of an individual who decides to take one drug placed on Schedule 1, perhaps without a medical prescription. This example only represents one side of the coin. To complement the analysis is necessary to consider another hypothesis, i.e., when one of these drugs (mainly a psychedelic) could be helpful to improve the health of this individual. However, if the current prohibition denies the medical prescription or the Law denies the treatment, the right to health is undermined by prohibitionist policies. However, self-medication is attached to a complex phenomenon such as self-harm. This fact is a good antidote against a naïve approach, which does not recognize the risks of medicalization.

The right to health is a core feature that must be considered in decriminalization efforts. Suppose a State cannot guarantee the right to health of its citizens due to the prohibitionist policies. In that case, it is necessary to promote a revision of the Law, to overcome the paradox of a failed State, which cannot guarantee the right to health and ensure freedom of choice. The right to health with psychedelic-assisted therapy (PAT) must have a universal character. However, this right to use psychedelics is usually denied with weakly grounded objections, which overshadows the core argument. Walsh (2010, 428) argued that the therapeutic use of psychedelics: “can be interfered with where ‘necessary’ in a democratic society in the interests of national security, public safety or the economic well-being of the country, for the prevention of disorder or crime, for the protection of morals.” A philosophical approach must be aware of these *ad hoc* objections to analyze them and propose solid counterarguments grounded in a culturally specific context.

In PAT, freedom of choice could be guaranteed when in the hypothetical situation of a clinical trial, a patient with depression can decide between treatment with anxiolytics (escitalopram) or psilocybin (Carhart-Harris et al., 2021, 1,403). Another clear example is when a patient with a terminal illness, such as cancer, decides to choose cannabis to relieve pain and psilocybin to face better the end of life; in this case, their rights must be guaranteed by the State and healthcare institutions (private or public). Furthermore, the right to die with dignity for patients with a terminal illness must be recognized and guaranteed. Another sensitive example in the USA is the right of war veterans with post-traumatic stress disorder (PTSD) to receive psychedelic-assisted therapy with MDMA or psilocybin. The goal is to relieve the psychological damage due to emotional traumas caused by war because the suicide rate in veterans is higher than that of civilians (Fox, 2018; Herrington, 2022). Moreover, the suicide rate among Native American teenagers is also higher than the national average in the USA (Calabrese, 2013).

### 2.2. Freedom of religion

Ritual and spiritual uses of psychedelics are at the core of the so-called psychedelic renaissance, mainly in areas such as anthropology and the history of religions. Both disciplines have contributed to a better understanding of ritual uses in ancient cultures, the spirituality of indigenous peoples, and new-age reinterpretations. Freedom of religion is one of the critical features of the so-called psychedelic research revival, mainly due to the implementation in the USA of the Religious Freedom Restoration Act (RFRA). This amendment has been very influential because it allows some exemptions from the general rule and mainly because it represents a chink in the armor of prohibition policies (Walsh, 2014). Very well-known examples in the USA are the Native American Church (NAC), some ayahuasca churches such as Uniao do Vegetal, Santo Daime church, and the Rastafari religion.

Establishing and recognizing NAC was arduous; even inside Native American peoples, it is possible to find histories of opposition and anti-peyotist movements. For example, “among the Navajos, the main barrier was not the Bureau of Indians affairs but rather the Navajo Tribal Council and anti-Peyotist Navajos acting independently. After the arrests of Peyotists priests in 1938, the Tribal Council met to discuss peyote in 1940” (Calabrese, 2013, 87). Also, the mass media played a significant role in the stigmatization and criminalization of NAC. There were many legal efforts to outlaw it, and many states passed laws against peyote during the first half of the 20th century. “However, thanks largely to its incorporation as a church (which began in Oklahoma in 1918), NAC survived and has struggled to defend the religious freedom of its members” (Calabrese, 2013, 89).

Nevertheless, the case that caused a dramatic shift within the cultural paradigm clashes was the *Employment Division of Oregon* v. Smith trial, 494 U.S. 872 (1990). Alfred Smith, a member of the Klamath Tribe, who was removed from his family and placed in a boarding school at eight years old, was employed by a substance abuse treatment facility in Rosemburg, Oregon. Paradoxically the director of the facility fired him because Alfred Smith was a member of the NAC and attended a peyote ceremony. Alfred Smith and Galen Black, who also was fired because he attended the ceremony, claimed unemployment compensation. The case reached the U.S. Supreme Court, which made its decision, about Native Americans without take into account the First Amendment. If peyote is illegal and Oregon could send Smith and Black to prison for using it, it could surely refuse to pay them unemployment compensation (Calabrese, 2013, 91).

In 1990 Anthonin Scalia ruled that exercising religious freedom contained in the First Amendment should allow law enforcement because religious pluralism was a “luxury” that could not be permitted. For example, an individual’s religious beliefs did not exempt anyone from compliance with a law prohibiting state-regulated conduct. With that ruling, the NAC lost the right to use peyote. This trial generated significant controversy and exposed a paradox since one of the reasons for the arrival of settlers in the USA was religious freedom. Paradoxically it is one of the country’s foundations, and that ruling took away the religious freedom of the Native Americans. The Supreme Court ruling triggered a movement to defend this civil right, as leaders of other religious organizations saw a threat to this right enshrined in the First Amendment. This movement led to the implementation of the Religious Freedom Restoration Act (RFRA). However, nowadays, peyote is considered Schedule I within the UN Convention on Psychotropic Substances (1971), i.e., without any therapeutic value. However, on the other hand, NAC considers it has therapeutic properties. Both sides of the coin clearly show one of the core traits concerning the cultural paradigm clash mentioned above.

Regarding the Global South, in Mexico, there is a legal exemption that allows the ritual and spiritual uses of psychedelics (mainly psilocybin mushrooms and peyote) among the indigenous peoples. At the beginning of 1940, just in the middle of World War II, the Mexican government decriminalized all drugs by publishing the *Reglamento Federal de Toxicomanía*s (Federal Drug Addiction Regulations). However, this initiative was ephemeral due to the international treaties signed and the pressure of Harry J. Anslinger (1892–1975), the first Commissioner of the Federal Bureau of Narcotics. The legal framework in Mexico does not lie mainly on the right of freedom of religion; instead, the legal framework is closer to the Customary Law System, a mild version of legal pluralism. Nevertheless, some ambiguity concerning legal frameworks for psychedelics prevailed in Mexican society. For example, “even though signing of the Vienna Convention on Psychotropic Substances in 1971, the Mexican government agreed to tolerate the ritual use of these substances by indigenous peoples.” (Dawson, 2015, 127). However, the Mexican government never passed legislation to this effect, and Mexican police regularly persecuted indigenous peyotists during these years.

Likewise, in the international arena, indigenous peoples usually face a lack of acknowledgment of their rights. Usually, freedom of religion is not guaranteed, and indigenous communities experience harassment by police forces, arbitrary imprisonment of their leaders and ritual specialists, territory displacements, and cultural extractivism. Some paradigmatic examples are the coca leaf among indigenous peoples in Perú and Colombia (Metaal, 2014, 25–45) or the ayahuasca ceremonies among the indigenous peoples from Brazil (Feeney and Labate, 2014, 111–130). Also, the hegemony of colonial views, which considers indigenous spirituality idolatry, witchcraft, or superstition, promotes intolerance and undermines religious freedom.

In addition to the examples mentioned above is necessary to argue that freedom of religion is in the Universal Declaration of Human Rights, the European Charter of Human Rights, and the United Nations Declaration of the Rights of Indigenous Peoples. In other words, freedom of religion is well established within International Law, but the controversies begin when psychedelics are considered sacraments. There is no standard criterion, and many court cases can be labeled as religious prosecutions. For instance, in the USA, even though the First Amendment guarantees freedom of religion, and the Supreme Court has cautioned lower courts, the reality is that:

The Supreme Court's amorphous definition of religion has not resulted in many successful defenses. Courts have employed various techniques to deny freedom of religion defenses, including simply concluding that a defendant’s claim to a religious use of illicit drugs is not sincere or credible. Under this latter approach, the court assumes that a religion incorporating drug use exists but then concludes the defendant does not truly believe it. With new and non-traditional religions, this technique has proven very effective (Brown, 2014, 49).

To examine the constitutive elements of cognitive liberty, it is also necessary to reflect further on freedom of religion, but from the other side of the coin. For example, many significant cases deserve attention, as mentioned above, but also because it is essential to address some frequently overlooked topics, especially regarding atheism, agnosticism, and skepticism. In this area, the psychedelic humanities are very useful in going beyond the cliches to challenge the discrimination of these minority groups, even inside the psychedelic community. For instance, only a few scholars (Letheby, 2022, 69–92; Glausser, 2021, 614; Walsh, 2010, 432–434) have considered atheism, but before addressing this intricate matter is essential to analyze the basic constitutive elements of self-determination.

### 2.3. Self-determination

Liberty has had its foundations since Ancient Greek Philosophy through the concept of *eleutheria*, translated as freedom. However, a careful analysis shed light on other constitutive elements; such terms include *autonomia* and *autarkeia* (self-sufficiency), but also *parrhésia* (freedom of speech), which designated above all relations between freedom and self-preservation and self-determination (Raaflaub, 2013, 8). Analyzing the links between self-determination and autonomy within the context of psychedelic assisted therapy requires further explanations. For instance, it is necessary to highlight how autonomy emerged as a political concept in Greece but evolved as one of the foundations of morals due to the internalization of its meaning by the rational consciousness, i.e., through the development of freedom of thought.

This “internalization” of autonomy by the rational consciousness is crucial to understanding its contemporary meaning and some paradoxes attached to it. Davis (2022, 87) provides a historical approach highlighting how autonomy emerged in Greece, which was associated with the citizen’s possibility to determine their *nomoi*, i.e., their constitution, way of life, and policies of their communities. Self-determination has also been considered a civil right, especially in the realm of International Law, and it involves sovereignty. In the political field, it is possible to reflect on collective self-determination. However, concerning psychedelic therapy, it is crucial to address individual self-determination because it relates to the configuration of identity and personality. Concerning its constitutive elements, “the concept of self-determination can be deployed in two ways: against other people who exercise alien determination over an individual, who thereby throws off the yoke of domination, or *vis-à-vis* non-human forces to expand one’s scope of action. The first instance shall be referred to here as *political self-determination* and the second as *technical self-determination*” (Fisch, 2015, 21).

Individual self-determination is crucial for cognitive liberty because it relates to mental autonomy. For example, in the field of psychedelic humanities, its meaning could be explained as the absence of interference, i.e., the right to keep individual thoughts, brain chemistry, mental autonomy, and personal data independent from government and companies, on the other hand, and as the right to enhance cognitive processes using neurotechnologies, i.e., psychedelic substances. Although the absence of interference (widely known as “negative liberty”) is a crucial feature within liberalism, it is necessary to update this due to the rapid development of neurotechnologies during the 20th century. In a broader sense, self-determination implies the recognition of the human consciousness to deliberate, judge, choose, and act between different ways of action, both in private and community life, i.e., collective self-determination, which involves freedom of choice and freedom of thought.

As previously mentioned, a critical approach to the role of autonomy and self-determination can open our view concerning PAT, especially regarding controversial topics such as mystical experiences, ego dissolution, and trivialization of the psychedelic experience. There are significant nuances that require further explanations. Moreover, in some cases is necessary to re-elaborate our frameworks to address sensitive issues frequently overlooked. For example, regarding the role of autonomy within PAT, Davis (2022, 94) argues that it would be convenient to consider the concept of autoheteronomy. However, what does this mean? Firstly, recognizing that the psychedelic experience could be understood as a sequence in which a heteronomous suggestion from the therapist (or therapy manual) during the preparatory phase could unleash its power of suggestion at the peak of the psychedelic experience. Secondly, to be followed in the integration phase, the patient is making autonomous or makes their own this experience. Both elements, the suggestion and reappropriation are part of the same process. Due to this, the oriented psychedelic experience could be considered as autoheronomy, i.e., an experience of the self as other and the other as self.

To overcome a mainly Euro-American perspective regarding collective self-determination is very important to consider a critical approach; the analysis of Jörg Fisch provides an insightful perspective. However, his approach is built mainly from a western perspective and does not adequately consider how it works the right to self-determination for indigenous peoples. Within psychedelic humanities, it is crucial because the right of self-determination allows the survival of the ritual uses of psychedelics inside indigenous communities for several centuries. Recent research carried out by indigenous scholars shed light on the role of indigenous self-determination within western psychedelic research. For instance, self-determination is considered the ground of some ethical principles, which are the core of indigenous liberty or sovereignty; both concepts lack acknowledgment. Self-determination is a sensitive issue concerning the regulation of tangible and intangible indigenous heritage, i.e., the intellectual property of traditional indigenous medicine. The legal development and recognition of indigenous traditional knowledge “must come from indigenous self-determined rules of law to ensure culturally sensitive policies” (Celidwan, et al., 2023, 6).

## 3. Philosophy of psychedelics

Nowadays is widespread to find in the mass media a large amount of news describing the advances of clinical trials or passionate claims supporting religious freedom. Although science and religion indeed contributed to changing the current paradigms and challenged the policies of punishment, it is also true that both do not cover all the possibilities and potential for cognitive liberty in the realm of psychedelic humanities. As mentioned above, freedom of thought is one of the constitutive elements of cognitive liberty. However, due to its enormous scope, it is necessary to develop a philosophical approach to go beyond the boundaries of religious freedom. So, the philosophical uses of psychedelics can contribute to a better understanding of consciousness, even if we use a metaphysical or a naturalistic framework.

An under-explored area is the philosophy of psychedelics, partly because philosophy is not very popular in the mass media, which covered the psychedelic renaissance, and because mainstream philosophy is permeated with dogmatism and internal colonialism inside academic institutions (González Romero, 2022, 77–94). Some philosophical uses of psychedelics have long been traced to the Western tradition until ancient Greece (Rinella, 2010; Sjöstedt-Hughes, 2016). However, since the second half of the 20th century, this issue started to be explicitly considered (Huxley, 1954/2004; Drake, 1965, 56–58; Osmond, 1971, 58–64). They all grasped the philosophical possibilities of psychedelics and made significant contributions. However, all these attempts were isolated lights in the darkness of punitive policy. It was until the 21st century that the research on this issue achieved a systematic development.

Currently is possible to find a diversity of perspectives. For instance, Letheby (2021, 24–27) analyzes mystical experiences in psychedelic therapy from a naturalistic approach. Naturalism is a valuable tool to challenge the “classical hypothesis” coined by Aldous Huxley that psychedelics mainly manifest a cosmic or Divine mind in an individual human mind. The question is, how can a finite being/mind know an infinite being/mind? Likewise, Peter Sjöstedt-Hughes analyses - grounded in the works of Benedictus Spinoza and Alfred North Whitehead- the role of panpsychism within the study of consciousness (2022, 211–236). Also, Hauskeller et al. (2022, 107–132), grounded in the framework of Frankfurt School critical theory, and Michel Foucault’s philosophy displayed a sharp analysis of power relations and paradoxes embedded in current clinical trials involving psychedelics.

However, what does it mean to discuss the philosophical uses of psychedelics? To answer this question, I would argue that it is necessary to challenge our shared beliefs and frameworks, put them aside, and make a *tabula rasa*. This shift is crucial to understanding that the psychedelic experience is a source of knowledge and not only a series of hallucinations and also encourages psychological insights. So, the starting point to understand the philosophical uses of psychedelics is to acknowledge the epistemic value of the psychedelic experience.

The next question is, what kind of knowledge is attached to psychedelic experiences? This question shows a challenging problem that requires patience and an interdisciplinary approach to address adequately. Nevertheless, it is possible to highlight the following features: (a) knowledge of the self, or *inner consciousness* (Weber, 2022, 249); (b) knowledge of the interconnectedness between human beings and nature due to the use psychedelic substances; in other words and ecological consciousness. Of course, these categories do not involve all the knowledge embedded in the psychedelic experience; they are only a glimpse of the full possibilities. For example, psychoanalysis and transpersonal psychology demonstrated that psychedelics explore and unlock the unconscious in therapeutic settings. The knowledge produced during the psychedelic experience is a vein of research that requires further exploration—primarily using a wide array of philosophical tools such as phenomenology, epistemology, ontology, esthetics, and bioethics.

Philosophy allows us to grasp naturalism and metaphysical frameworks; due to this, it is possible to appreciate both sides of the psychedelic experience regarding therapeutic uses. On the one hand, naturalism represents an excellent antidote to metaphysical sickness, i.e., romanticizing spiritual hallucinations and denying the material content of psychedelic experience (Letheby, 2021). On the other hand, metaphysics could help go beyond a naïve realism embedded in a dogmatic view of western biomedicine. Sjöstedt-Hughes (2022) highlighted the therapeutic properties within metaphysics and the lasting psychological benefits of mystical experience. However, to achieve a better understanding, a systematic analysis of the metaphysical inputs and their social and cultural background is an issue that deserves attention in future research.

Philosophy is crucial to develop rigorous frameworks regarding a topic that requires exceptional accuracy to avoid hasty generalizations, cliches, and misunderstandings. Recognizing that the psychedelic experience is a source of knowledge, not a series of hallucinations, is the first step to overcoming the prevalent stigma (Letheby, 2016, 2021). Philosophy has demonstrated how the psychedelic renaissance is trapped in a false dilemma between religious and therapeutic uses of psychedelics, i.e., between hospitals and churches. This narrow approach represents a serious obstacle to achieve an understanding of the full potential of psychedelics (González Romero, 2022, 82). Acknowledging philosophical uses of psychedelics opens the doors of perception to different realms regarding creativity, self-reflection, psychological insight, and connectedness with nature.

### 3.1. Inner consciousness

The knowledge attached to psychedelic experience is related primarily to the knowledge of the self, which involves psychological insights, meditation processes, and reflective attitudes. All of them can produce dramatic transformations in personal beliefs, behaviors, and even in the worldview of individuals (Letheby, 2021, 53–61; Timmermann et al., 2021, 1–13; Nayak et al., 2022, 1–13). The common feature shared between philosophy and psychedelics is that both are useful for expanding consciousness, as mentioned above. The awareness of inner consciousness is closely related to one of the constitutive elements of cognitive liberty, i.e., self-determination.

Due to this, the more explicit philosophical uses of psychedelics are attached to this topic. For example, some common expressions found in the literature are altered states of consciousness (ASC), non-ordinary states of consciousness (NOSC), and modified states of consciousness (MSC). They have advantages and disadvantages, but I argue that the former is accurate for avoiding ambiguities and characterizing the knowledge and transformation embedded within psychedelic experiences.

The concept of consciousness is not free from ambiguities; other terms such as soul, mind, spirit, or personality have been used inaccurately as synonyms. Moreover, unlocking the unconscious is a crucial feature of psychoanalysis as a therapy and could be considered a type of knowledge. For instance, some frameworks, such as the one developed by Carl Gustav Jung, underline the significance of the collective unconscious (Jung, 2005/1933). Also, the approach developed by transpersonal psychology encourages the dissolution of the ego (Grof, 2012, 137). Moreover, transpersonal psychology proposed a cartography of the psyche related to psychedelic experiences.

An analysis grounded in naturalism can explain the neurocognitive processes underlying the lasting psychological benefits of psychedelic therapy and allows us to go deeper in our understanding of consciousness. The contribution of philosophy also lies in the fact that it provides a coherent explanation of the links between self-narratives and self-representations with essential brain networks such as Default Mode Network (DFM) and Salience Network (SN) (Letheby, 2021, 93–101). Undoubtedly, philosophy is a handy tool to extend our knowledge regarding consciousness from non-clinical uses of psychedelics. Philosophy stimulates freedom of thought and critical analysis of the psychedelic market.

Summarizing, the knowledge provided by psychedelic experience could produce subjective changes and meaningful transformations, which are vital constitutive elements in self-determination. Inner consciousness is a core component of free will. The knowledge of the self is an unfinished process, and oriented psychedelic experiences can contribute to achieving some improvements, as clinical trials and psychotherapy have been demonstrated (Letheby, 2021, 62–79). A philosophical approach can better explain PAT’s mechanism and achieve a sharp interplay between science and humanities.

### 3.2. Ecological consciousness

A widespread feature within contemporary debates considers that the psychedelic experience enhances connectedness between human beings and nature. Psychedelics can be understood as a booster to achieve this ecological consciousness faster than any other way because they provide a vivid experience of this connection. However, to make the most of it, a sharp reflection on the frameworks will be beneficial; for example, from a naturalist approach, those feelings of connectedness could be explained through a connectivity ontology, which is one of the key features that come from environmental humanities.

Of course, there are other frameworks to explain this phenomenon; for instance, the most common are attached to mystical experiences or spirituality (Timmermann et al., 2021, 1–13). Despite its popularity, those metaphysical frameworks imply many assumptions and are filled with a cultural background everyone does not share. Due to this, a connectivity ontology allows us to more accurately explain the ecological consciousness and the knowledge achieved during the psychedelic experience rather than some metaphysical inputs.

According to the interdisciplinary frameworks provided by psychedelic humanities, these studies on ecological consciousness pretend to explain the relationship between human beings and nature with a different framework than the hegemonic paradigm, which prevails in western philosophy and science. “The traditional separation between those disciplines concerned with ‘nature’ and those that examine ‘culture’ has led to increasingly atomized science-based responses to environmental dilemmas” (O’Gorman et al., 2019, 427–460). In the context of the transformation caused by climate change, the psychedelic humanities and indigenous philosophies represent an alternative to strengthened ecological consciousness with a cross-cultural perspective.

To understand the value and contributions of indigenous philosophies is necessary to consider seriously an ontological turn (Holbraad and Pedersen, 2017, 1–29) or, in other words, a shift in the hegemonic paradigm which prevails nowadays. Furthermore, critical reflection on the traditional boundaries between nature and culture (Descola, 2013, 3–32) can explain some cultural traits embedded within indigenous philosophies, which are not evident at first glance. The bridge between psychedelic humanities and indigenous knowledge is the notion of ecological interconnectedness, i.e., the assumption that the human being is part of an extensive living system that does not separate mind and body, nature and culture, science and humanities, or organic and inorganic life (Rose, 2005, 294). This connectivity ontology is the key feature to building bridges of understanding between psychedelic humanities and ecological consciousness.

### 3.3. The doors of perception and the ontological turn

What kind of knowledge is attached to opening the doors of perception? To answer this question is crucial to carry out a philosophical interpretation of Aldous Huxley’s proposal. For instance: “when the doors of perception are opened, the Aristotelian logic is revoked and its ontological counterpart- substance ontology relativized” (Weber, 2022, 253). Of course, according to Aldous Huxley, the opening of the doors of perception has a larger scope and allows us to see or understand things that are not evident in our ordinary states of consciousness. Furthermore, regarding contemporary philosophical terms, the consequence of this opening is an ontological turn. This ontological turn, understood as a heuristic device, helps to grasp different worldviews if the philosophical mind embraces an intercultural approach.

Opening the doors of perception involves the acknowledgment of a connectivity ontology and an interdisciplinary theoretical marker to face some environmental dilemmas concerning climate change, preservation of biodiversity, rights of non-human species, food sovereignty, water management, and pollution. Indigenous philosophies and western ecological thinking come from different sources but have some features and concerns in common. Due to this, it is possible to talk about an indigenous philosophical ecology (Rose, 2005, 294). From an anthropological approach, the central concern of the ontological turn is “It is about creating the conditions under which one can see things in one’s ethnographic material that one would not otherwise have been able to see” (Holbraad and Pedersen, 2017, 4).

Beyond western tradition, indigenous philosophies contribute to extending the scope of psychedelic humanities. Firstly, they encourage the ontological turn better to comprehend the cultural significance of the psychedelic experience (Williams et al., 2022, 506–527). Secondly, indigenous philosophies can clarify the ontology of connectedness between human beings and nature but without succumbing to anthropocentrism. Thirdly, this ontological turn is a clue to strengthen the framework of psychedelic humanities and overcome the ambiguities embedded in humanism. For instance, indigenous philosophies recognize the significance of the More-than-Human (MTH) beings regarding the knowledge embedded within the psychedelic experience.

Robert Warrior, an Osage scholar, identifies *topos* (territory or place) as foundational to indigenous philosophies in contrast to *logos* (discourse or the word) which underpins Western philosophy (Williams et al., 2022, 510). This shift is not only theoretical; it is related to sensitive political issues attached to the right to land and ecological consciousness. From a materialist perspective, this cultural feature is handy in explaining the significance of the land and the connectedness between humans and plants, fungi, animals, rivers, lakes, lagoons, seas, forests, and glaciers. Pointing out this cultural feature is very important because the psychedelic renaissance and western academia tend to overlook the material character of indigenous philosophies to focus on spiritual and metaphysical features. Moreover, indigenous philosophies are mainly a way of living and not only a discourse produced in seminars, books, or papers.

Regarding consciousness, some noteworthy developments embedded within the ontological turn include the notions that forests think (Kohn, 2013, 131–153), that rivers are persons (Hutchison, 2014, 179–182), and that plants are intelligent (Kimmerer, 2015; Gagliano, 2018). All these topics require an independent study but shed light on the scope of ontological pluralism concerning the psychedelic experience. For instance, contemporary researchers are interested in how psychedelics may produce a wide range of significant transformations in perception, cognition, and mood. A recent study explored belief changes related to psychedelic experiences, which are deeply associated with qualitative features (Nayak et al., 2022, 1–13). The research results were organized into five factors: 1.Dualism; 2.Paranormal/Spirituality; 3.Nonmammal consciousness- refers to whether insects, trees, and rocks are capable of having a conscious experience and 4.Mammal consciousness.

Resetting how human beings build their links with nature and society is a crucial topic for psychedelic humanities. Moreover, the transformation produced regarding self-representation and ecological consciousness could strengthen the meaning and scope of the possible emancipatory uses of psychedelics. Of course, to avoid a naïve perspective, it is necessary to recognize that psychedelics are not the solution to all problems and illnesses. Psychedelics are not magic bullets; it is also crucial to encourage meaningful transformations within the “global set and setting” and the social causes of depression, anxiety, addictions, and PTSD (Hendlin, 2022). To go deeper is necessary to argue that a complete transformation of the global “set and setting” requires a shift in how the psychedelic experience is conceived within the margins of capitalism and mainstream mass media. For example, a lack of investment in education, health, housing, and transportation, will inevitably lead to a mental health crisis and cannot be addressed by a therapeutic approach alone. Enhancing the frameworks and methodologies applied in contemporary research is crucial to open the doors of perception to a broader scope.

The psychedelic humanities allow us to extend the scope of consciousness into a broader framework, not restricted only to biological or individual processes but also consider their social connections (Roberts, 2017, 102–105). The transformations attached to self-narratives are not isolated from a global “set and setting,” which shape and determine some of the cultural features, restrictions, or pathologies everyone must deal with (Hendlin, 2022). The connection between individual self-narratives, oral history, and social narratives is an issue that deserves further research (O’Gorman et al., 2019, 284). The psychedelic humanities represent a suitable and well-equipped laboratory to explore these overlooked connections and account for the self-determination processes regarding identity, citizenship, and gender.

The ontological turn is necessary to acknowledge indigenous philosophies’ value and scope. Indigenous peoples have a millenary knowledge of ritual, divinatory and therapeutic uses of psychedelics. The consequence of this turn is the recognition of ontological pluralism, which helps to overcome the paradoxes and colonial shadows embedded in the psychedelic renaissance (Negrin, 2021, 65–70). Ontological pluralism is not only a scholarly matter but also a crucial feature in better understanding some social issues. For example, struggles for territory, epistemological extractivism, and the patent system (Gerber et al., 2021, 573–577). Within indigenous worldviews, peyote and sacred mushrooms are not considered mainly psychedelic substances isolated from the territory but also living beings with whom it is possible to establish communication through ritual and ceremonial language.

The ontological turn is not without controversy, and there is an extended debate about its foundations and scope. Some scholars argue it could imply an anachronistic extrapolation of western frameworks to indigenous worldviews (Course, 2010, 247–263). Others consider it embraces a meta-ontology, i.e., a meta-narrative, making it challenging to understand the phenomena it seeks to explain (Heywood, 2012, 146). Furthermore, it has been argued that “ontology” is only another word for culture (Carrithers et al., 2010). On the other hand, supporters used to consider that the ontological turn is simply a technology of description, which allows anthropologists to make sense of their ethnographic materials (Pedersen, 2012). However, the ontological turn is not only circumscribed to the indigenous worldviews or cosmologies, but its scope also embraces the fields of ‘cyberdelics’ and ‘technodelics’, especially concerning the challenges attached to a posthuman realm (Hartogsohn, 2023, pp. 1–8). This reflection about the role of the human, posthuman, and more than human entities (MTH) is at the core of the psychedelic humanities because, despite its limitations, the ontological turn could be a useful heuristic device to overcome dogmatism.

## 4. The psychedelic humanities

Roberts (2017) and Langlitz (2019) highlighted the “sorry state” of humanities at the beginning of the 21st Century. Technocratic regimes dismissed the role of humanities in education, preferring the natural sciences, cybernetics, and business as the core education. However, the mainstream turn involved in the psychedelic renaissance represents the opportunity to shift this technocratic paradigm to achieve a holistic overview to overcome the fictitious split between science and humanities, mind and body and subject-object. Humanities and decolonial theory help avoid manichean dualisms and cultural biases within psychedelic studies (Hauskeller et al., 2022).

The development of psychedelic humanities implies a shift of social, cultural, and scientific paradigms, a complete substitution of frameworks and methodologies (Fanon, 2004/1961). A decolonial approach implies, a complete substitution of the punishment policies which undermined freedom of choice and caused social paradoxes such as violence, murders, forced disappearances, imprisonments, racism, and discrimination (González Romero, 2022). A decolonial approach will be helpful to understand from a broader perspective the social contradictions attached to the policies of punishment, but also regarding psychedelic capitalism.

The psychedelic humanities are interdisciplinary, combining historical texts, community-based narratives, anthropological fieldwork, and contemporary scientific claims. It draws on diverse methodologies from history, ethnography, hermeneutics, literary theory, and quantitative-qualitative methodologies, including interviews and surveys (Roberts, 2017, 102–119; Langlitz, 2019, 275–288). By comparing philosophical projects across time and place, psychedelic humanities explore how indigenous knowledge has intersected (or not) with western claims of cognitive liberty within a clinical encounter or a therapeutic model. Based on community-engaged approaches and historical, anthropological texts, psychedelic humanities can generate a sophisticated analysis of how prohibition efforts altered the philosophical goals of psychedelic use.

### 4.1. History

Historical research is crucial in overcoming biases produced by the ideology behind the prohibition policies. The propaganda used at the beginning of the war on drugs undermined the legitimacy of scientific research on psychedelics, but also disseminated cultural biases, racialization and discrimination of psychedelics use among the general audience. Moreover, this strategy shared false information and fake news without enough scientific evidence. A clear example was the anti-drug scare film “LSD 25,” launched in 1967. Unfortunately, this propaganda dismissed the potential therapeutic uses of LSD to treat alcoholism based on the research carried out by Humphry Osmond and Abram Hoffer (Dyck, 2008, 13–32).

Another example was the misinformation regarding the chromosome-damaging, carcinogenic, and mutagenic effects attributed to LSD consumption. For instance, *Science News* Vol 91, 1 April 1967, published a brief note called “LSD may damage chromosomes” the note highlighted those two researchers from the State University of New York tested LSD on cell cultures of the blood of two healthy individuals. “The New York study published in *Science*, March 1967 indicates that in addition to psychosis, LSD can produce biological damage at the most basic level.”

Many examples in newspapers, TV, and comics spread this propaganda and misinformation. However, further scientific research did not support these statements. “From our own work and from a review of the literature, we believe that pure LSD ingested in moderate doses does not damage chromosomes *in vivo*, does not cause detectable genetic damage, and is not a teratogen or a carcinogen in man” (Dishotsky et al., 1971, 431–440).

Due to this, the role of psychedelic humanities lies, in fact, in providing a rigorous revision of this propaganda based on the evidence of scientific and historical research. The psychedelic humanities could overcome misinformation, racialization, and propaganda attached to prohibitionist policy. For instance, a war on intellect is the collateral damage of the war on drugs and the propaganda attached to it (Roberts, 2017, 116). The revisionism of the war on drugs includes assessing and evaluating past experiences, incidents, and ideologies to provide an adequate framework to support the therapeutic purposes of current clinical trials and create a free cultural environment from biases and misinformation.

On the one hand, to avoid a naïve perspective is essential to highlight that revisionism can serve definable political, ideological, or cultural ambitions used for legitimization purposes. On the other hand, revisionism can encourage the rise of historical consciousness to avoid past mistakes and bad practices or redefine group or national identities. Historical consciousness is a specific type of collective and social consciousness but also with an individual character. Both paradigms (collective and individual) are frequently overlapped, and psychedelic humanities can contribute to disentangling its scope:

[…] the first one considers historical consciousness as a collective phenomenon and studies its perceived rise as a pivotal moment in the genesis of modern self-understanding; the second one treats historical consciousness as an individual competence and uses it for the training of cognitive capacities with which people can understand the past (Grever and Adriaansen, 2019, 815).

Regarding the first paradigm, i.e., historical consciousness as a collective phenomenon, critically revisiting the war on drugs can enhance cognitive liberty within academic institutions and mass media. Moreover, to produce and promote high-quality research on psychedelics and the implementation of public policies which can guarantee human rights (Walsh, 2010). The second paradigm helps strengthen the cognitive capacities of those individuals who use the psychedelic experience as a psychological insight and for therapeutic purposes without fear or remorse.

History allows a better understanding of psychedelics’ diversity and cultural uses. This acknowledgment of diversity is essential to avoid the hasty generalizations and dogmatism embedded in the prohibitionist policy. Pluralism and diversity are core features within psychedelic humanities, especially regarding non-western worldviews. For example, history and anthropology are fundamental to encouraging reconciliation and reciprocity with indigenous peoples.

### 4.2. Anthropology

Psychedelic humanities must consider the ontological turn explained above to overcome some paradoxes attached to the meaning of a key concept: “humanism.” With the help of anthropology, it is necessary to explain the differences between psychedelic humanities and psychedelic humanism. Regarding this issue, Langlitz (2020b) argues that the main difference is that psychedelic humanities do not attribute prime importance to humans rather than the divine or other beings such as animals or plants; in other words, psychedelic humanities do not embrace anthropocentrism.

To establish a clear difference between both, Langlitz (2020b) analyzed the failure of humanism, especially regarding its narrow scope. To achieve its goal challenged a core belief within humanism, i.e., that only human beings share certain features such as consciousness, agency, choice, responsibility, and morality, even though these capacities can be lost in particular circumstances. In a broader sense, psychedelic humanities can contribute to encouraging the reflection of what it means to be a human being within the Anthropocene and to enhance critical thinking concerning the impact on ecosystems caused by human activities. The rise of ecological consciousness is critical to understanding the scope of oriented psychedelic experiences.

Anthropology has the potential to play a substantial role in promoting new knowledge that works within psychedelic and environmental humanities exploring nature in terms of agency and communication, i.e., moving beyond the hegemonic paradigm of anthropocentrism, which prevails in Western science and philosophy. Plumwood (2002, 8) proposed two significant tasks regarding this issue. “The first is to re-situate the human in ecological terms, and the second is to re-situate the non-human in ethical terms.” Oriented psychedelic experiences can contribute to a better understanding of both tasks, not only in a theoretical way but also with a hands-on approach. The psychedelic humanities are a laboratory in which it is possible to systematically analyze both perspectives (indigenous philosophies and environmental humanities) to develop better frameworks to understand the connectivity ontology between human beings and nature under the influence of psychedelic substances.

### 4.3. Critical thinking

Critical thinking is valuable for shaking the grounds of mainstreaming psychedelics and revealing the paradoxes embedded in psychedelic capitalism, which is a topic frequently overlooked by enthusiastic and naïve approaches (Hauskeller et al., 2022). An epistemic virtue ethics allows a deeper understanding of cognitive freedom’s foundations, scope, and limits (Langlitz, 2019, 284). Ethics promotes a critical approach to the ideologies within psychedelic science and market, i.e., the medicalization of psychedelics (Noorani, 2019, 34–39), the patent system (McGonigle, 2016, 217–226), and cultural appropriation (Gerber et al., 2021, 573–577). Developing decolonial ethics will help face some of the unexpected dilemmas attached to psychedelic science and the opening of new markets. The accessibility of the new therapies developed is a social issue that must be considered seriously.

The significance of psychedelic humanities in encouraging critical thinking; this task could be achieved in two ways. Firstly, on the one hand, an historical revision of the war on drugs is necessary to overcome the stigma, discrimination, and murders caused by several decades of punishment policies. Secondly, disseminating the outputs based on scientific research to general audiences is the first step to providing psychedelic education. Prohibitionist policies attached to the war on drugs undermine cognitive liberty and civil rights in four ways:

1. Are a severe threat to the freedom of thought.
2. Are a denial of the right to health.
3. Imply the lack of acknowledgment of indigenous rights.
4. Represent an obstacle to self-determination and the freedom of choice.

Each of these topics requires an independent assessment. However, all of them must be considered for decriminalizing drugs and developing public policies based on harm reduction and the management of pleasure. Secondly, on the other hand, to avoid a simple-minded approach regarding psychedelic capitalism (Hallifax, 2023), it is necessary to display a critical analysis of the power relations, economic interests, and political agendas embedded in the psychedelic renaissance. There is a long history of bioprospecting for gain in the Global North where psychedelic compounds and plants are seen to have epistemic and financial value. The psychedelic humanities are far from a purely enthusiastic approach. Trivialization and monetization can undermine the emancipatory uses attached to the psychedelic experience, i.e., a transformation of the self-narratives, the rise of ecological consciousness, the right to cognitive liberty, the dissolution of authoritarian values and beliefs, among others.

Critical thinking would help better understand the emancipatory potential of psychedelics and the transformative processes regarding the self. “The psychedelics are a red-hot, social/ethical issue precisely because they are de-conditioning agents. They will raise doubts in you if you are a Hassidic rabbi, a Marxist anthropologist, or an altar boy because their business is to dissolve belief systems” (Forte, 1997, 61). On the other hand, this is only one side of the coin; to avoid a naïve perspective is necessary to acknowledge that they also have significant reconditioning potential: as pluripotent enhancements of suggestibility and non-specific amplifies. People have the right to refrain from using neurotechnologies and deserve protection from coercive and unconsented administration, because psychedelics are also strong conditioning agents. Ethics in clinical trials and PAT must be aware of free, prior, and informed consent to avoid unwilling administration of psychoactive substances, and to ensure ethical data management.

A significant critical thinking issue is recognizing the risks and limitations of the spiritual market and clinical colonization, to overcome some paradoxes and achieve a synergy between science and the humanities. For example, some paradoxes become visible in the following questions: is it legitimate to encourage the consumption of an endangered cactus (peyote) or animal (*bufo alvarius* toad) to satisfy the “spiritual needs” of the tourists or churches? Or is it legitimate to undermine the emancipatory potentials of psychedelics to transform them into a commodity controlled by pharmaceutical companies?

The spiritual market attached to the psychedelic renaissance is another topic that deserves further study because religious or spiritual uses are one of the legitimated uses because they are under the umbrella of religious freedom. Firstly, it is necessary to be aware of the side effects of neurotheology, because some religious organizations are behind the scenes of some studies related to the change in metaphysical beliefs (Glausser, 2021, 614). Secondly, it is necessary to recognize that some vegetal species, such as peyote, *bufo alvarius* toad and the components of ayahuasca, are endangered due to overexploitation attached by churches and neo-shamanic movements. Thirdly, it is necessary to recognize the psychological abuses, sexual harassment, and psychological violence attached to some psychedelic sects (Sánchez, 2019). Due to this, the psychedelic humanities could be a handy tool to address many social issues appropriately beyond clinical and therapeutic uses.

Critical thinking in the psychedelic humanities must also avoid exoticizing and romanticizing indigenous cultures. Regarding exoticization, it is crucial to underline that cultural appropriation and lack of respect are two features that must be considered carefully; in that sense, philosophy and anthropology can adequately address these issues. Concerning romanticization, developing “god-savage ethnographies” promotes an idealistic representation, which overlooks the conflicts and struggles faced by indigenous peoples. Moreover, most anthropologists, philosophers, and historians need professional knowledge of indigenous languages to understand indigenous philosophies fully. This lack of knowledge of indigenous languages is the cause of the arbitrary appliance of Western categories and frameworks, such as shaman, healer, sorcerer, magician and witch, among others. The irreflexive use of these categories could lead to an *epistemicide* of indigenous knowledge. To avoid some paradoxes, the psychedelic humanities must encourage the implementation of linguistic criteria.

According to a decolonial approach (Tuhiwai, 2008), a complete transformation of International Law is required to reschedule some sacred plants and fungi used by indigenous peoples since several centuries ago. Also, to guarantee the rights of indigenous peoples, encourage the preservation of endangered plants and cacti, especially peyote, and develop a fair patent system based on legal pluralism (McGonigle, 2016; Celidwen et al., 2023). Furthermore, to be coherent with critical thinking, it is necessary to recognize the possible risks regarding psychedelic-assisted therapy, the limitations of current clinical trials, which does not fully recognize the indigenous knowledge, and the inequalities embedded in the development of psychedelic capitalism.

A major ethical issue is spreading truthful information to overcome the stigma and disinformation attached to the policies of prohibition. Due to this fact, psychedelic humanities can contribute to achieving a balance and finding more accurate categories and frameworks considering ontological pluralism and cultural diversity. For example: “Euro-American misunderstandings and assumptions about “drugs,” with the prototype of “drug use” in the Euro-American imagination perpetually reduced to the figure of a socially dysfunctional heroin addict or drunk escaping from reality (Calabrese, 2013, 82–83). Psychedelic humanities can contribute to clarifying these hasty generalizations and developing a more accurate framework.

Another relevant topic is discussing if the war on drugs is effectively ending or if the psychedelic renaissance is just a global redistribution of prohibitionist territories and techniques. Analyzing the constitutive elements of psychedelic capitalism is necessary to address this topic adequately. In legal terms, it is possible to appreciate a mild transformation of legal frameworks and public policies, especially regarding cannabis and psychedelics. Acknowledging therapeutic and adult uses allows appreciating a change in the mid-term, i.e., in two to five years. This shift in the prohibitionist paradigm could be explained due to the interest of pharmaceutical companies in opening new markets. The pharmaceutical industry has traditionally opposed decriminalizing drugs and psychoactive substances, but it seems that nowadays is interested in a new market. However, to avoid a naive perspective, it is too soon to consider that the war on drugs is ending. Nowadays, we are experiencing mainly a redistribution of the markets. The possible therapeutic advantages developed by psychedelic science are not available to most human beings. Furthermore, non-religious and adult uses still are stigmatized and prosecuted.

Summarizing, some of the substantial contributions of psychedelic humanities could be listed as follows:

1. Medical anthropology and linguistics allow the strengthening of methodologies regarding transcultural psychiatry and ethno-psychiatry.
2. Philosophy and history of religions are helpful for better understanding the “mystical experience.”
3. History allows a better understanding of the diversity of cultural uses that psychedelics have had over time.
4. Philosophy allows understanding that therapeutic and spiritual uses are not the only valid uses for psychedelics.
5. Law and sociology help design better public policies and regulatory frameworks.
6. Art and literature allow for widening the scope of psychedelic-assisted therapy.
7. Pedagogy allows the creation of educational programs, didactic resources, and materials on the advantages and risks of psychedelics for different population sectors.

## 5. Conclusion

Cognitive liberty involves at least four philosophical meanings: (1) freedom of choice, (2) freedom of religion, (3) self-determination, and (4) freedom of thought. This paper focused on explaining its constitutive elements, but of course, further research can contribute to addressing this topic’s scope and social implications. Freedom of thought requires special attention, and due to this, the section on the philosophy of psychedelics was devoted to clarifying some challenging topics. The main one is to explain the core features regarding philosophical uses of psychedelics; this issue is relevant because, within the psychedelic renaissance, the main legitimate uses are therapeutic and spiritual. However, critical thinking is essential to avoid getting trapped in the false dilemma between hospitals and churches or new-age psychedelic sects and conspiracy theories. Due to this, it is necessary to consider a philosophical approach seriously. The first step is recognizing that the psychedelic experience is a source of knowledge and not only a series of hallucinations. The second step is to face the question, what kind of knowledge is embedded within the psychedelic experience?

According to the findings of this research, it is possible to highlight (a) knowledge of the self, or *inner consciousness*, and (b) knowledge of the interconnectedness between human beings and nature, or *ecological consciousness*. Psychedelic knowledge goes beyond psychologism or ideology and includes knowledge of the brain and changes in beliefs. Nevertheless, it also includes the ontological turn and developing critical thinking through freedom of thought. Of course, there are multiple ways to achieve a philosophical approach. Langlitz underlined the possibilities within neuro-philosophy and the perennial philosophy. However, it is also necessary to consider indigenous philosophies contributions seriously, especially because historical and archeological evidence demonstrated the use of psychedelic plants and fungi several millennia ago.

Historical research is crucial in overcoming cultural biases and racialization produced by prohibition policies. History helps carry out a revisioning of the war on drugs, mainly because critical revisionism can encourage the rise of historical consciousness to avoid past mistakes and bad practices or redefine group or national identities. Historical consciousness contributes to moving from psychologism, subjectivism, and relativism. Furthermore, history acknowledges women’s work in psychedelic research, which is frequently overlooked due to colonialism and structural violence. The history of psychedelic science is a vein of research that helps better understand the social processes attached to the interplay between science and humanities.

Concerning the role of anthropology, it is necessary to highlight that psychedelic humanities do not attribute prime importance to humans rather than the divine or other beings such as animals or plants; in other words, psychedelic humanities do not embrace anthropocentrism. For instance, this is the main difference between psychedelic humanities and psychedelic humanism, which must be recognized to avoid misunderstandings. Considering the ontological turn could help strengthen the reconciliation processes with indigenous peoples. Furthermore, anthropology can encourage the reflection of what it means to be a human being within the context of ontological pluralism. Medical anthropology could involve fieldwork inside the laboratories and health institutions where psychedelic science and clinical trials are developed and within indigenous medicine and traditional healing systems.

The humanities contribute to developing innovative and rigorous frameworks to carry out psychedelic research that integrates critical thinking including decolonizing approaches, equity, diversity, and inclusion strategies, and alternative perspectives drawn from social justice claims concerning the potential benefits of psychedelics in contemporary societies. The role of psychedelic humanities is to encourage dialog and freedom of thought and to produce uncomfortable questions. Self-criticism prevents against simple-minded approaches and challenges the idealization of psychedelic science, clinical trials, patent systems and indigenous worldviews.

The entire development of the humanities could strengthen the education regarding psychedelics in larger audiences due to the lack of education provided by nation-states, private companies, and NGOs globally. In spite the fact of some significant efforts, the politics of punishment still prevail worldwide. The development of psychedelic education will contribute to facing some mental health challenges. Also, education will help develop harm reduction policies to alleviate the unnecessary suffering triggered by the war on drugs. A trans-disciplinary approach within psychedelic research can help to overcome the “sorry state” of the humanities attached to neo-liberal and technocratic regimes to fully recognize that philosophical uses of psychedelics can help to broaden the mental landscapes regarding cognitive liberty and encourage critical thinking to overcome the attempts to control and manipulate human consciousness.

## Data availability statement

The original contributions presented in the study are included in the article/supplementary material, further inquiries can be directed to the corresponding author.

## Author contributions

The author confirms being the sole contributor of this work and has approved it for publication.

## Funding

This research has been supported by the University of Saskatchewan, through the Miseweskamik International Postdoctoral Fellowship.

## Conflict of interest

The author declares that the research was conducted in the absence of any commercial or financial relationships that could be construed as a potential conflict of interest.

## Publisher’s note

All claims expressed in this article are solely those of the authors and do not necessarily represent those of their affiliated organizations, or those of the publisher, the editors and the reviewers. Any product that may be evaluated in this article, or claim that may be made by its manufacturer, is not guaranteed or endorsed by the publisher.
